# Factors associated to the career choice of family medicine among Japanese physicians: the dawn of a new era

**DOI:** 10.1186/s12930-014-0011-2

**Published:** 2014-10-03

**Authors:** Kenya Ie, Masao Tahara, Akiko Murata, Manabu Komiyama, Hirotaka Onishi

**Affiliations:** 1 Department of Family Medicine, Mie University School of Medicine, Mie, Japan; 2 Naniwa Clinic, Amagasaki Health Cooperative, Hyogo, Japan; 3 Family Practice Center of Okayama, Okayama, Japan; 4 Shonan Sanada Clinic, Kanagawa, Japan; 5 International Research Center for Medical Education, Graduate School of Medicine, the University of Tokyo, Tokyo, Japan

**Keywords:** Career decision making, Health policy, Medical education, Medical students, Multivariate predictive models, Primary care

## Abstract

**Background:**

Despite recent developments in post-graduate family medicine training in Japan, the numbers of junior doctors entering family medicine residencies are still limited. The objective of this qualitative study was to investigate the possible factors associated to the career choice of family medicine, especially in the context of the newly established family medicine programs in Japan.

**Methods:**

From December 2010 to January 2011, we distributed a semi-structured questionnaire about career choice to 58 physician members of the Japan Primary Care Association, and 41 of them responded. Four researchers used the Modified Grounded Theory Approach (Kinoshita, 2003) for three-stage conceptualization.

**Results:**

We extracted a conceptual model of the choice of newly established family medicine as a career in Japan, consisting of six categories and 77 subordinate concepts from 330 variations. The subcategories of personal background affecting the family-medicine career choice were characteristics (“self-reliance,” “pioneering spirit”), career direction (“community/rural-orientedness,” “multifaceted orientation”) and experience (e.g., “discomfort with fragmented care”). We divided the influencing factors that were identified for career choice into supporters (e.g., “role model”), conflict of career choice (e.g., “anxiety about diverse/broad practice”), and the dawn of a new era in family medicine in Japan (e.g., “lack of social recognition,” “concern about livelihood,” and “too few role models”).

**Conclusions:**

Although the dawn of a new era seemed a rather negative influencer, it was unique to our study that the dawn itself could attract those with a “pioneering spirit” and an “attitude of self-training.” Unlike previous studies, the positive factors such as lifestyle and the short residency program were not shown to be part of family medicine's attractiveness. In contrast, “concern about livelihood” was specific among our respondents and was related to career choice in the dawn period. “Community-orientedness” and “multifaceted orientation” (which have aspects in common with previous studies' findings) would appear to be universal regardless of cultural and medical system differences. In our study, these universal factors were also found to be part of the attractiveness of family medicine from the practitioners' viewpoints, and these factors may become great influencers for family medicine candidates.

**Electronic supplementary material:**

The online version of this article (doi:10.1186/s12930-014-0011-2) contains supplementary material, which is available to authorized users.

## Background

Japan has the highest life expectancy in the world [1]. Some unique features of the Japanese healthcare system may contribute to better health outcomes and thus to the long life expectancy. First, the Japanese government has provided universal health insurance coverage since 1961. Second, patients in Japan have the privilege of free access to healthcare without any formal gatekeeping system.

In most of the countries in which a healthcare system is well established, healthcare roles are clearly divided between the provision of primary healthcare by general practitioners, and the provision of secondary to tertiary care by specialists. The Japanese healthcare system, in contrast, has not established any definite division between general practitioners and specialists. In addition, no formal postgraduate training program for family medicine was developed in Japan until 2006.

Most of the conventional primary care physicians in Japan were originally trained as specialists; typically, after completing specialty training and subsequent work experience as a hospital-based specialist for some years, they open their own private clinic without any formal family medicine training. Additionally, Japanese society's increasing demand for quality healthcare has raised a new issue pertaining to the lack of formal training for primary care physicians in Japan [2],[3]. Before the merger of the Japanese Society of Primary Care, the Japanese Academy of Family Medicine (JAFM) and the Japan Society for General Medicine as the Japan Primary Care Association (JPCA) in 2010, the JAFM had implemented a three-year family medicine program for certified physicians since 2006.

In 2012, the Japanese Ministry of Health, Labour and Welfare announced a new board certificate system for physicians' specialties by a third-party organization starting in 2017. Along with this reform, “General Practice” including family medicine has formally become the 19th new specialty. Although the need for formally trained family physicians in Japan is increasing, our preliminary study (2011 unpublished) showed that only 1% of junior doctors are entering the JPCA certified family medicine residency program. To enhance the recruitment of family medicine residents, it is essential to understand the factors that influence the choice to practice family medicine versus other fields of medicine.

In the United States and Canada, aside from a dip in the late 1990s, the number of family physicians has increased significantly over the last few decades. Despite this positive trend, the numbers of residents choosing the family medicine specialty remain well below those nations' needs. Studies from Europe and North America revealed several common factors associated with the career choice of family medicine. In a review article, factors that were positively associated with the choice to practice family medicine were “rural background”, “parents' low socioeconomic status”, “low interest in research career” and “planning on a career in a disadvantaged or rural area” [4]. Negative factors were “concerns about prestige”, “low income”, and “breadth of knowledge required” [4]. “Low intellectual content” was also suggested as a reason for rejecting family medicine [5].

The American Academy of Family Physicians (AAFP) devised a Student Interest “Influencer” Portfolio [6] to explore medical students' interests as documented in four regional Student Interest Stakeholder meetings in 2010 and 2011. The AAFP held the meetings to counteract the stagnation in students' interest in practicing family medicine. The meetings showed that the positive perceptions of family medicine include “diverse and complex patients (never bored)”, “family and life-friendly specialty”, “highest recruited specialty since 2007” and “specialty for all environments/settings practice options.” Negative perceptions, on the other hand, were “too easy/too hard paradox”, “scope of practice paradox (only colds and coughs)”, “doesn't make any money”, “life style — can't do it all (take calls, deliver babies, etc.)”, “bashing from different specialties”, and “medical school — lack of exposure to family medicine”.

In Japan, there have been a few reports about the factors associated to the career choice of “conventional” primary care/rural medicine. For example, toward the goal of increasing the number of primary care providers, Ohtaki et al. proposed that the medical education system in Japan must provide both primary care doctors to act as role models and readily accessible information about postgraduate primary care programs [7]. Ae et al. found that residents who expected to engage in rural practice were significantly more likely to choose primary care as a future career, and residents with training experience in a rural setting had a significantly higher tendency to change careers from a specialty to primary care [8]. Thus, Ae et al. noted that exposure to rural practice during residency training can affect residents' career plans. However, to our knowledge no report on career choices with respect to the newly established family medicine specialty in Japan has been published.

The objective of the present qualitative study was to investigate the possible factors associated to the career choice of the newly established family medicine program in Japan.

## Methods

### Design

A qualitative study using semistructured questionnaire was conducted.

### Data collection

From December 2010 to January 2011, four family medicine researchers (authors KI, MT, AM and MK) sent invitations to participate in this study to 58 physician members of the JPCA via email; 41 (70.0%) responded. Written consent was obtained from those who agreed to participate in the study. Since no database regarding physicians in the family medicine field was established at the time of this survey, all respondents were personal acquaintances of the four researchers. Of the 41 respondents, 35 (85.0%) were men. The respondents' mean length of experience as a physician was 8.2 years (range 3-20 years). The respondents' main practice setting was a clinic for 30 (73.2%), a hospital for 7 (17.1%), and an academic medical center for 4 (9.6%).

The questionnaire was semi-structured and based on a framework invented by group discussion of four researchers (KI,MT,AM and MK) following a literature review. We lined up questions we believed necessary. Then all questions were reviewed and rewritten if the item was ambiguous or unnecessary. It included the following four questions:

1. How and when did you first experience family medicine? Tell us about your personal first encounter with family medicine.
2. Why did you decide on family medicine as a career choice? Tell us in terms of your own personal experience, motivation, and ideas.
3. Have you had any obstacles or conflicts to choosing family medicine as a career? If so, what were they?
4. What do you think about the appeal of family medicine?

### Analysis

The four researchers used the Modified Grounded Theory Approach (Kinoshita 2003 [9]), developed from the Grounded Theory Approach proposed by Glaser and Strauss in 1967 [10]. The questionnaire results were independently read by research team members to identify key variations. Following the initial reading, an analytical work sheet was used as the basis for conceptualization and categorization. First, concepts were generated by the research team from the variations. Next we made categories from several concepts to produce a three-stage conceptual model. A medical education supervisor (author HO) repeatedly reviewed the study process and the conceptual model for triangulation.

## Results

We extracted 77 subordinate concepts from 330 variations. We had set the framework of this study as “family medicine career choice”. Eight concepts were excluded from the conceptualization for two reasons; 1) responses were not specifically related with family medicine (e.g. resistance to detachment from hometown), and 2) responses were beyond our scope of the research (e.g. issues raised after the career was chosen).

**Figure 1:**
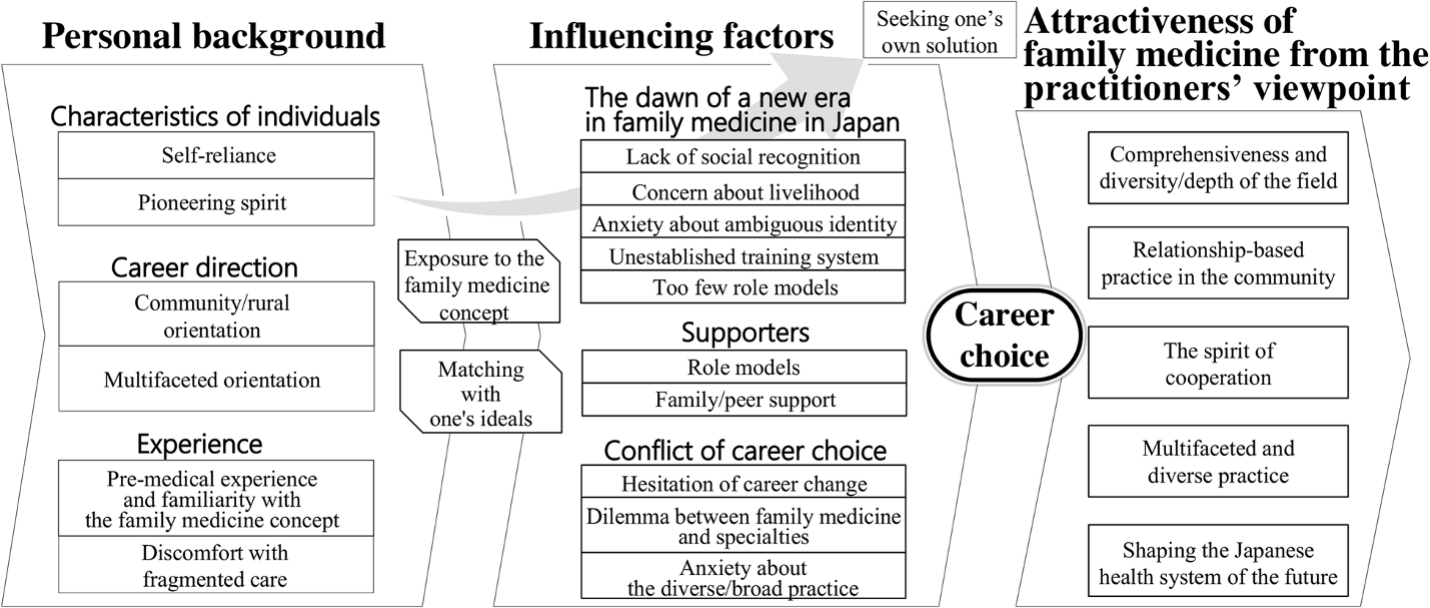
**The process of choosing family medicine as a career.**

The conceptual model consisted of six categories: (a) personal background, (b) exposure to the family medicine concept, (c) matching with one's ideals, (d) influencing factors, (e) seeking one's own solution, and (f) attractiveness of family medicine from practitioners' viewpoints (Figure 1).

### a) Personal background

#### Characteristics of individuals

Characteristics which affected the choice of family medicine consist of the following two concepts.

##### Self-reliance

A self-reliant personality not affected by surroundings was one of the common characteristics among the respondents. As one respondent put it:

*“I thought that every obstacle would be no surprise, and paying my full attention to surmount the obstacles would be a worthwhile job.”*

##### Pioneering spirit

The other characteristic of note was the pioneering spirit of those who found the frontier field of family medicine worthwhile:

*“I expected to be a pioneer and felt excited, because I was certain that family medicine was not yet widely known in Japan.”*

#### Career direction

Two major directions for the choice of family medicine as a career were “community/rural orientation” and “multifaceted orientation”.

##### Community/rural orientation

*“I wanted to be engaged in a doctorless village. I thought family medicine was most suitable for the needs of such a community.”*

*“Family physicians can stand on the same ground with patients and community residents. It allows me to be myself. That's why I wanted to be a family physician.”*

##### Multifaceted orientation

Many respondents were interested in many fields of medicine and wanted to deal with the entire realm of medical practice. Some respondents initially chose a specialty rather than family medicine but were familiar with a multifaceted orientation; they had not had the opportunity to know family medicine.

*“When I became a doctor, I thought I wanted to be a doctor who could deal with any kind of physical problem.”*

#### Experience

Experiences that strongly influenced the family medicine career choice were “pre-medical experience and familiarity with the family medicine concept” and “discomfort with fragmented care”.

##### Pre-medical experience and familiarity with the family medicine concept

For some of the respondents, the choice of family medicine as a career was based on personal experience: when a family member became ill, the respondent's pre-medical experience with the primary care physician who cared for the family member was a strong influence. Other respondents had career directions aligned with family medicine from a psychosocial standpoint, such as influence by a teacher or a clinical psychotherapist.

*“A member of my family became bedridden due to a stroke. When the caregiving had exhausted other family members, I felt the thoughtfulness of the home care doctor was splendid.”*

##### Discomfort with fragmented care

Some respondents had uncomfortable feelings toward the highly specialized and disease-oriented medicine usually seen in large-scale hospitals.

*“When an internist saw a patient with pneumonia, the internist simply said 'Pneumonia is not my specialty.' I was really disappointed.”*

### b) Exposure to the family medicine concept

At various times and in a variety of settings, all of the respondents had been exposed to the family medicine concept although the conventional medical education in Japan offers no official opportunity for learners to be exposed to family medicine. Lectures or seminars outside the university or textbooks were alternative opportunities. After the respondents' exposure to family medicine, there were various first impressions.

*“When I first had contact with the family medicine concept, I felt 'This is it!!'”*

*“I felt dubious about the family medicine concept and not sure if it could be a lifelong career.”*

### c) Matching with one's ideals

Some of the respondents felt that family medicine matched with their ideals, and they started thinking of family medicine as a career specialty.

*“I was impressed at family physicians' role in helping citizens, families and the community. My intuition told me that family medicine was my life's work.”*

### d) Influencing factors

In the process of choosing family medicine as a career specialty, several important factors had both positive and negative influences.

#### Supporters

Respondents noted that the existence of family physician role models and empathic support from their family and colleagues were indispensable promoters of the family medicine career choice.

##### Role models

Some respondents had a family member who was a primary care physician, and other respondents stated that a primary care physician cared for an ill member of his or her family. In each case, the primary care physician was a role model for the respondent.

*“A family physician who cared for me and my mother has been my role model. So I was thinking about being the kind of physician who can deal at first with any problem.”*

##### Family/peer support

Empathic support from family and colleagues and the increasing social status of family physicians had positive influences on the respondents' career choice.

*“I had a feeling that I wasn't isolated, because of the increased social recognition and increased family medicine applicants among medical students.”*

*“When my wife told me, 'I guess you are suitable for a family physician,' I was really encouraged.”*

#### Conflict of career choice

##### Hesitation of career change

The respondents who had practiced a specialty other than family medicine tended to hesitate when it came to re-training for family medicine.

*“I felt as if I had to lose my original specialty to select family medicine as my career.”*

##### Dilemma between family medicine and specialties

Some respondents reported facing a dilemma between the choice of family medicine and another specialty when they were interested in family physician.

*“I was unable to make up my mind whether to continue pediatrics or to change my career to family medicine.”*

##### Anxiety about the diverse/broad practice

*“I didn't have confidence that I could deal with such a broad and general field.”*

#### The dawn of a new era in family medicine in Japan

As noted earlier, a three-year family medicine program was started in Japan in 2006, and when the present study's questionnaire was compiled, there were no more than approximately 200 certified family physicians in Japan. In addition, only 1% of junior doctors are entering the family medicine residency program. The main factors that had a negative influence on the choice of family medicine as a career among the present study's 41 respondents were “lack of social recognition”, “concern about livelihood”, “anxiety about an ambiguous identity”, “unestablished training system” and “too few role models”.

##### Lack of social recognition

Lack of understanding from individual around the respondents had a negative impact on the career choice of family medicine.

*“No one around me understood family medicine as a career specialty, so I was not sure if I could do it.”*

*“During my clinical clerkship, some teaching staff scoffed at my career dream to be family physician, and said, 'Such a specialty doesn't exist (in Japan).' ”*

##### Concern about livelihood

*“I felt insecure about my future career and my family's livelihood because family medicine was not acknowledged as a specialty at that time.”*

##### Anxiety about an ambiguous identity

Low social recognition of the family medicine field made it difficult to establish an identity.

*“When asked about my specialty, especially what family medicine is, I couldn't really answer....”*

##### Unestablished training system

Lack of a family medicine program, colleagues, and training information were obstacles for the respondents who were interested in family medicine.

*“When I was a junior resident, only a few training facilities and colleagues were acknowledged. I even thought of going to the U.S. for family medicine training.”*

##### Too few role models

The respondents had seldom observed a family medicine role model because the number of role models was quite limited.

*“I couldn't imagine what I would be like after gaining experience as a family physician.”*

### e) Seeking one's own solution

Some respondents had a strong will to solve or surmount the problems/obstacles associated with family medicine training by themselves.

*“There were very few family medicine training facilities at that time. So I started to gather information about training facilities and visited some of them.”*

### f) Attractiveness of family medicine from the practitioners' viewpoint

After the respondents chose family medicine as a career, many of them were satisfied with their jobs. The following five family medicine-specific attributes were found to be the most attractive after the respondents had chosen family medicine as a career.

#### Relationship-based practice in the community

Some respondents felt that having a continuous relationship with their patients was an appealing aspect of family physicians. The concept of “Relationship” implied an ongoing relationship with patients or the community and other healthcare providers.

*“I think I can provide compassionate medical treatment by taking good care of not only the disease but also the lives of the patients and people.”*

*“Family physicians can build up a team in a rural community that eases people's minds and enables large-scale jobs which no one can carry out by themselves. This is an aspect of family medicine that appeals to me.”*

*“Being with patients, families and the community as a family physician has expanded my perspectives.”*

#### The spirit of cooperation

Some respondents described cooperativeness, flexibility and educational passion as appealing aspects of family medicine.

*“I think there is very good environment in this field where we value our peers, and there is not any infighting.”*

#### Multifaceted and diverse practice

Respondents reported that family medicine is attractive because it does not limit one's field but provides various healthcare options for various people.

*“In a way, every patient I see is within my field. Besides, understanding patients' familial/social contexts can improve the quality of care.”*

#### Shaping the Japanese health system of the future

Some respondents regarded the possibility of solving healthcare problems and developing a new academic field in Japan to be appealing.

*“Academic aspects of family medicine impressed me because forerunners have been verbalizing and deepening the areas not previously well conceptualized in Japan. Furthermore, the aspect of family medicine implies not only a conceptual framework but also practical aspects.”*

#### Comprehensiveness and diversity/depth of the field

The practice of family medicine consists of both broad and deep aspects of all of the fields of medicine.

*“The broad field of family medicine is worth spending the rest of my whole life learning.*

## Discussion

This qualitative study aimed to investigate the possible factors associated to the career choice of family medicine and those associated to enrollment in the newly established family medicine program in Japan. The process of choosing a career in family medicine is multifactorial and complex. Several factors appear to be characteristics of the choice to go into family medicine practice. Those factors can have positive or negative influences on the career choice of family medicine.

In Japan at this stage, there are not enough family physician role models or family medicine curricula/residency programs. Previous studies outside of our country describe encounters with family physician during postgraduate training or in medical school curricula [11],[12], but in our study, most respondents' exposure to the family medicine concept was incidental, by way of books or seminars. The responses from participants in this study indicated that medical students and residents who had never known the concept of family medicine did not choose family medicine specialty even if they had felt a “discomfort with fragmented care”.

Although the respondents noted several negative influences on the choice of family medicine as a career (i.e., lack of social recognition and of a guarantee of livelihood, the absence of role models and training systems, and anxiety about an ambiguous identity), the factors of “pioneering spirit” and “self-reliance” can be strong accelerating influences in this newly established field. In addition, the feeling that a new era is dawning in family medicine in Japan may contribute to the choice of family medicine as a career. Some candidates may be attracted by “seeking one's own solution” that family medicine may still require in Japan. In a description of physicians in North America and the UK who chose family medicine, Taylor wrote they were self-confident, visionary, audacious, passionate, and energetic [13].

In our study, unlike previous findings, the factors of lifestyle [11],[14],[15] and short residency program [11],[16] were not shown to be indicative of family medicine's attractiveness. In contrast, we found that “concern about livelihood” was a specific concern in the dawn of the new family medicine era. Achieving career security and/or a guarantee of income seems to be difficult in the present circumstances and thus a pioneering spirit and a sense of self-reliance may be critical to a candidate's choice of family medicine as a career.

Actual clinical family medicine exposure that demonstrates enduring relationships with patients, continuity of care, and preventive medicine are essential to attracting candidates to family medicine [11]. Several common factors such as the amount of exposure to family medicine, existence of a family medicine department and a residency program in his/her medical school have been described as accelerators for the family medicine career choice [11],[12],[15]. Thus, increasing the amount of exposure to family medicine is expected to lead more young physicians to select family medicine in Japan.

Family medicine's “community/rural orientation” as described in the present study has aspects in common with the known premedical attributes familiar to family medicine (humanitarian outlook and rural background) [6],[11],[16]. We also found that family medicine's “multifaceted orientation” is a point of appeal that may be aligned with some physicians' preference for the varied tasks of family physicians described in previous studies [6],[14],[17]. These present and previous results imply that there are several universal orientations among family medicine candidates regardless of cultural or medical system differences. In our study, these universal factors were also found in attractiveness of family medicine from the practitioners' viewpoints, and thus the constructive presentation of these attractive points may positively influence candidates considering the practice of family medicine.

Not only amount of exposure to family physicians, but also existence of good role models could greatly impact on family medicine candidates [11],[18]. Medical school faculty members who can convey the attractiveness of family medicine as a career could also make a significant contribution to this impact [11].

### Study limitations

The semi-structured questionnaire that we used could lead each response in a certain direction. The relatively short work experience of the respondents (mean 8.2 years) might have produced a lack of continuity, academic issues and education in the attractiveness category. In addition, the generalizability of these research findings is limited because they were generated in an exploratory qualitative inquiry. It was difficult to avoid potential sampling or recall bias.

## Conclusions

It was unique to our study that the dawn itself could attract those with a “pioneering spirit” and an “attitude of self-training”, although the dawn of a new era seemed a rather negative influencer. The known positive factors such as lifestyle and the short residency program were not shown to be part of family medicine's attractiveness in our study. In contrast, “concern about livelihood” was specific among our respondents and was related to career choice in the dawn period. These dawn-specific factors clarified by this research could contribute to the development of family medicine in countries which have introduced the same changes as Japan. Further studies are needed to confirm whether the characteristics of the choice to practice family medicine identified here will continue to play a role in the future. Moreover, following studies to clarify the reasons why some physicians did not choose family medicine specialty could be useful.

## Authors’ original submitted files for images

Below are the links to the authors’ original submitted files for images. Authors’ original file for figure 1
