# Supplementary figures and images for: Factors associated to the career choice of family medicine among Japanese physicians: the dawn of a new era
Source: Asia Pac Fam Med. 2014 Oct 3;13:11. doi: 10.1186/s12930-014-0011-2 (PMC5377022; doi:10.1186/s12930-014-0011-2)

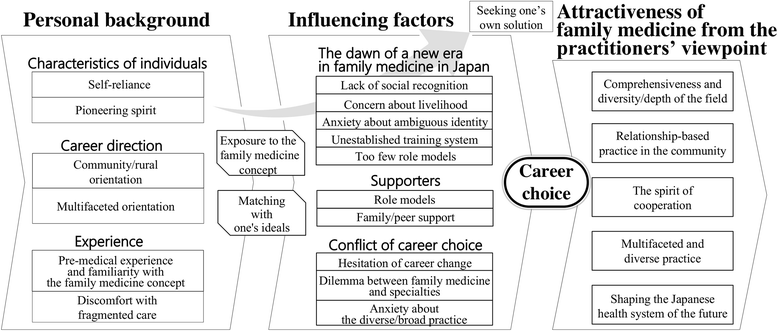

Supplement: Supplementary file 1 — Authors’ original file for figure 1 [file 12930_2014_11_MOESM1_ESM.gif]
